# Influence of skeletal muscle heterogeneity on autophagic signaling and response

**DOI:** 10.1080/27694127.2025.2562429

**Published:** 2025-10-08

**Authors:** Fasih A. Rahman, Joe Quadrilatero

**Affiliations:** Department of Kinesiology and Health Sciences, University of Waterloo, Waterloo, ON, Canada

**Keywords:** Skeletal muscle, autophagy, heterogeneity, fiber type, muscle plasticity, metabolic phenotype

## Abstract

Skeletal muscle is a heterogeneous tissue composed of fibers with distinct contractile, metabolic, and molecular characteristics. This intrinsic heterogeneity influences how individual fibers respond to physiological stimuli, pathological stress, and cellular remodeling processes such as autophagy. Skeletal muscle autophagy is essential for maintaining proteostasis and organelle quality, particularly in high-demand tissues like skeletal muscle. However, emerging evidence indicates that autophagy is not uniformly regulated across all muscles and fibers within a skeletal muscle. Fast/glycolytic fibers, characterized by faster contractile speed and high glycolytic capacity, exhibit greater autophagic flux potentially driven by activation of energy signals, calcium, and redox-sensitive pathways. In contrast, slow/oxidative fibers, characterized by slow contractile speed and higher oxidative metabolism, show lower inducible autophagy despite elevated basal expression of autophagy-related proteins. These differences are compounded by fiber type – specific organelle architecture, recruitment patterns during activity and disuse, and substrate availability and utilization. Further, pathological conditions such as disuse, chronic disease, and myopathies often induce fiber type alterations as well as changes to organelle content and function that are closely associated with changes in autophagy signaling. Additionally, species and strain variability add another layer of complexity, complicating both the interpretation and translational relevance of autophagy studies in skeletal muscle. This review synthesizes current evidence linking skeletal muscle phenotype to autophagy regulation and highlights the need to consider skeletal muscle heterogeneity as a central variable in skeletal muscle autophagy research. A deeper understanding of skeletal muscle type/fiber-specific autophagy will improve our ability to interpret experimental findings and develop targeted interventions for skeletal muscle dysfunction.

## Introduction

Skeletal muscle is a remarkably versatile tissue that plays a central role in locomotion, metabolic regulation, endocrine signaling, and systemic homeostasis^[1,2]^. Despite its broad physiological significance, it is often underappreciated in discussions of complex biological processes, particularly those related to cellular remodeling and stress responses. One of the most defining characteristics of skeletal muscle is its intrinsic heterogeneity. Traditionally, skeletal muscle fibers have been categorized based on their contractile protein isoforms into fiber types such as type I (slow-twitch) and type II (fast-twitch). However, this classification only partially reflects the functional complexity of skeletal muscle tissue. An additional and equally important classification is based on metabolic phenotype, which reflects a fiber’s capacity for energy production and substrate utilization. Metabolic phenotype is shaped by factors such as organelle content/density and the expression of metabolic enzymes, all of which influence a fiber’s ability to respond and adapt to physiological stimuli. Further adding to this complexity, skeletal muscle fibers exist along a continuum of cellular and molecular properties, and can dynamically shift their phenotype in response to various stressors and perturbations such as exercise, disuse, or disease. In this context, neuronal input and motor unit recruitment patterns play a major role in shaping skeletal muscle phenotype, as different fiber types/motor units are selectively recruited depending on force demands and task specificity^[3–5]^. These recruitment patterns influence intracellular signaling cascades, organelle function, and molecular signaling, ultimately guiding how a skeletal muscle fiber adapts^[6–10]^. Together, the layered regulatory inputs converge to shape distinct cellular signaling responses under various physiological contexts. Importantly, these intrinsic differences also shape how skeletal muscle responds to disease which often disproportionately affect specific fiber types or metabolic states, disrupting normal signaling, and impairing remodeling processes^[11–25]^. Moreover, unpublished data from our group and others suggest that biological variability across species and genetic backgrounds adds further complexity to our understanding of cellular signaling processes and their implications on skeletal muscle biology^[26–28]^. Differences in fiber type distribution, recruitment patterns, and signaling responses between species or strains can significantly impact how experimental results are interpreted. This highlights the need for a nuanced approach in the study of skeletal muscle biology, where heterogeneity is not treated as a confounding variable but rather as a feature to be understood. Recognizing and integrating this heterogeneity is essential for advancing our understanding of skeletal muscle biology and resolving inconsistencies in the literature.

**Figure 1.**
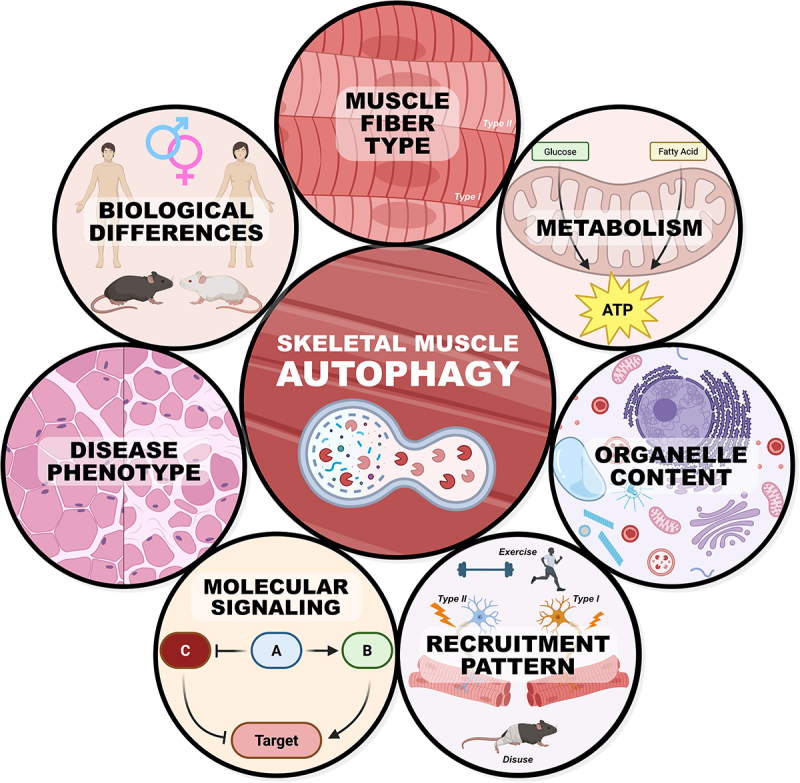
Conceptual model illustrating the multifactorial regulation of autophagy in skeletal muscle. This figure provides a visual overview of key factors that influence autophagic activity in skeletal muscle. Autophagy is dynamically regulated by intrinsic properties such as fiber type composition, metabolic phenotype, organelle content/morphology, and recruitment patterns during activity or disuse. Additionally, molecular signaling pathways modulate autophagic flux in a fiber type- and context-dependent manner. External factors such as disease state, species, or strain differences further contribute to observed differences in skeletal muscle autophagy.

Growing interest in skeletal muscle biology has led to greater investigation into the role of autophagy within skeletal muscle. Autophagy is a fundamental catabolic process essential for maintaining cellular homeostasis through the targeted degradation and recycling of damaged organelles, aggregated proteins, and other cytoplasmic constituents via the lysosomal pathway. In the context of skeletal muscle, autophagy facilitates fiber remodeling and adaptation in response to a wide range of physiological and pathological stimuli. However, given the intrinsic heterogeneity of skeletal muscle, it is becoming more evident that select forms of autophagy (e.g., mitophagy) are not uniformly regulated across all skeletal muscles, fibers, or metabolic phenotypes (unpublished observations)^[29]^. Emerging evidence indicates that autophagic flux and regulatory mechanisms are influenced by multiple factors, including fiber type, metabolic phenotype, organelle content, and recruitment pattern. Furthermore, species variation and genetic background introduce additional layers of complexity that can influence key cellular parameters, such as oxidative capacity and mitochondrial content, which in turn may modulate autophagic activity^[27,28]^. Importantly, the autophagy-specific consequences of these features are not yet fully understood and remain an area for future investigation. As such, understanding the regulation of autophagy in skeletal muscle necessitates an appreciation of this biological heterogeneity. Studies that fail to account for skeletal muscle heterogeneity may overlook fiber type-specific or context-dependent differences that influence autophagic dynamics. In this brief review, we synthesize current knowledge at the intersection of skeletal muscle heterogeneity and autophagy, with particular emphasis on how differences in skeletal muscle phenotype and molecular patterns shape the regulation and functional consequences of autophagy (Figure 1). We aim to provide a broad framework for understanding how autophagy contributes to skeletal muscle plasticity and the maintenance of tissue integrity across diverse physiological and disease contexts.

## Limitations of MYH-based fiber classification and implications for autophagy regulation across muscles

Skeletal muscle fibers have traditionally been classified based on the expression of specific myosin heavy chain (MYH) isoforms, which correlate with the contractile speed and mechanical properties of each fiber^[30]^. Early classifications were informed by differences in myofibrillar ATPase activity at varying pH levels^[31,32]^, and later refined through immunohistochemistry and transcriptomic profiling to distinguish MYH isoforms with greater precision^[26,33,34]^. Muscle fiber types can broadly be classified as type I (slow-twitch) or type II (fast-twitch) which reflects their contractile speed. In humans, type II fibers are further subdivided into type IIA and type IIX, with contractile velocity increasing from type I to IIX (i.e., I < IIA < IIX). In commonly studied animal models like rodents, an additional even faster fiber, type IIB, is also present, but absent in human skeletal muscle. Although this framework remains widely used, its limitation is in the ability to capture the full phenotypic range of skeletal muscle fibers, particularly with the recognition of hybrid fibers that co-express multiple MYH isoforms^[26,35]^. Moreover, fiber type distribution varies across individual human skeletal muscles. For example, the vastus lateralis (VL), commonly used in human studies due to its accessibility, displays a relatively heterogeneous fiber type profile that broadly reflects the average MYH distribution seen in skeletal muscle^[36]^. This reliance on a single muscle group may bias our broader understanding of skeletal muscle or fiber heterogeneity. Notably, MYH-defined fiber types do not always align with metabolic phenotype or contractile function^[26,35]^. Similarly, assumptions that fiber size or force output correlate directly with MYH expression can be misleading^[37]^. A more integrative classification system that considers both molecular and functional attributes is needed to more accurately capture the complexity of skeletal muscle biology and to improve translational relevance across experimental models. In the context of autophagy, rodent models remain central due to the accessibility of multiple skeletal muscle groups, whereas most human studies tend to be limited to the VL^[38–40]^. Nevertheless, increasing evidence indicates that autophagy is not uniformly regulated across skeletal muscles with different fiber type composition. In rodent skeletal muscles such as the soleus (SOL) and diaphragm (DIA), which are enriched in type I and IIA fibers, there is consistently higher basal expression of key autophagy-related proteins including autophagy-related 7 (ATG7), ATG12, and beclin 1 (BECN1) when compared to muscles composed mainly of type IIX and IIB fibers like the white vastus lateralis (WVL), white quadriceps (WQ), and plantaris^[41–43]^. In contrast, other proteins involved in autophagy initiation, such as unc-51 like autophagy activating kinase 1 (ULK1) and ATG5, are more abundant in skeletal muscles like the tibialis anterior (TA) and gastrocnemius-plantaris complex (GP), which have a higher proportion of IIX and IIB fibers^[44]^. While downstream markers like microtubule associated protein 1 light chain 3 (MAP1LC3 or LC3) are generally more prevalent in skeletal muscles like the SOL and red quadriceps (RQ), the distribution of sequestosome 1 (SQSTM1) appears less consistent across studies^[41,43,44]^. Proteomic analyses of isolated human skeletal muscle fibers also suggest fiber type-specific regulation of autophagy. LC3B levels were found to be highest in type IIX fibers, followed by type IIA and type I fibers^[34]^. However, basal protein expression does not always reflect dynamic autophagic activity. In vivo autophagy flux experiments in mice using colchicine (a microtubule polymerization inhibitor that blocks autophagosome-lysosome fusion) have shown greater LC3B-II accumulation in predominantly type IIX and IIB muscles (e.g., TA, GP, EDL) compared to predominantly type I and IIA muscles (e.g., SOL, DIA), suggesting that autophagic flux may be elevated in muscles with a faster contractile phenotype^[43,44]^. Similar patterns are observed during fasting, where LC3-II levels increase more rapidly and robustly in predominantly type IIX and IIB muscles (e.g., TA, GP, and EDL) compared to predominantly type I and IIA muscles (e.g., SOL and DIA) of mice^[44,45]^. Collectively, these observations underscore that while skeletal muscle fiber type is a key determinant of skeletal muscle phenotype, it does not fully explain the complexity of autophagy regulation. This is due in part to the uncertainty around whether observed differences are directly attributable to fiber type itself, or instead to other correlated properties such as metabolic profile, organelle content, or recruitment patterns. A deeper understanding of this heterogeneity will be essential for elucidating the context-specific mechanisms governing autophagy across different muscle environments.

## Influence of metabolic specialization of skeletal muscle fibers on autophagic responses through substrate availability and energetic stress

As alluded to earlier, skeletal muscle fiber types differ not only in their contractile and metabolic properties but also in their intrinsic energy storage and utilization capacity. To reflect the functional and metabolic specialization of skeletal muscle fibers more accurately, we will refer to them hereafter as fast/glycolytic and slow/oxidative, based on their predominant contractile velocity and metabolic phenotype. While these classifications provide a useful framework for distinguishing broad functional differences, it is important to recognize that they are generalizations, as skeletal muscle fibers exist along a continuum with substantial overlap in metabolic and contractile properties both within and across skeletal muscles. Fast/glycolytic fibers are characterized by substantially greater glycogen content (ranging from 16% to 31% higher than their slow/oxidative counterparts) in humans, reflecting their reliance on anaerobic glycolysis for rapid ATP production during high-force contractions^[46,47]^. In contrast, slow/oxidative fibers are enriched in intramyocellular lipid droplets compared to fast/glycolytic fibers (7 mM vs. 4.2 mM; 0.5% vs. < 0.1% of fiber volume)^[48,49]^, and possess greater mitochondrial volume in humans (6% in type I fibers, compared to 4.5% in type IIA and 2.3% in type IIX fibers)^[49]^, supporting sustained fatty acid oxidation (FAO) and aerobic ATP production at rest and during prolonged contractile activity. These distinct substrate storage profiles shape metabolic function under both resting and exercise conditions. At rest, slow/oxidative fibers primarily oxidize fatty acids, maintaining a stable ATP/ADP ratio and redox balance, which dampens activation of energy sensors such as AMP-activated protein kinase (AMPK)^[50]^. In contrast, glycolytic fibers, despite their higher glycogen stores, remain less metabolically active and exhibit minimal carbohydrate turnover at rest^[51,52]^. However, during high-intensity exercise, these fibers rapidly deplete their glycogen reserves^[48,51]^, triggering energetic stress, a drop in ATP, and subsequent AMPK activation which is a known inducer of autophagy^[53]^. Supporting this, autophagic flux, measured using colchicine, was significantly elevated in the fast/glycolytic TA muscle from mice both immediately post-exercise and after 90 minutes of recovery^[54]^, consistent with glycogen utilization as a trigger for autophagy activation. In contrast, slow/oxidative fibers maintain ATP production via ongoing FAO and thus experience less energetic perturbation during endurance activity^[55,56]^. This is consistent with the findings showing that slow/oxidative muscles (e.g., SOL and RQ) from mice exhibit lower autophagy flux despite higher basal expression of some autophagy proteins including ATG7 and BECN1^[41–44]^. Supporting this, a negative linear correlation between LC3B-II flux and citrate synthase (CS) activity (i.e., a marker of oxidative capacity) has been reported in mice under both basal and fasted conditions with fast/glycolytic muscles like the TA and GP complex exhibiting lower CS activity but higher LC3B-II flux, whereas slow/oxidative muscles such as the SOL and DIA display the opposite pattern^[44]^. Nevertheless, the availability of metabolic substrates including carbohydrate or fatty acids and their metabolic intermediates can also modulate autophagic signaling. For instance, glycogen depletion activates AMPK, thereby promoting autophagy, whereas glycogen accumulation appears to impair autophagy^[11,18,57,58]^. Similarly, FAO increases the NAD/NADH ratio, leading to activation of sirtuin 1 (SIRT1), which enhances autophagy through deacetylation of proteins such as forkhead box O (FOXO) family of transcription factors and LC3^[59]^. On the other hand, high levels of acetyl-CoA can suppress autophagy by promoting protein acetylation and inhibiting autophagy-related gene expression^[60]^. These opposing effects illustrate how fluctuations in key metabolic intermediates can dynamically regulate autophagic flux and link substrate utilization to cellular remodeling capacity in a fiber and context-dependent manner, particularly as they interact with recruitment patterns. However, the extent to which these metabolic-autophagy interactions vary between different skeletal muscles/fibers, each with distinct contractile properties and substrate preferences, remains poorly understood and warrants further investigation.

## Organelle specialization and autophagic regulation in slow/oxidative and fast/glycolytic skeletal muscles

Skeletal muscle fibers exhibit differences in organelle composition that align with their functional demands for force generation, endurance capacity, and metabolic specialization. Among the most extensively studied are the sarcoplasmic reticulum (SR) and mitochondria, though differences may also extend to other organelles such as peroxisomes, lysosomes, and nuclei^[33,61–63]^. In the context of muscle type classification, fast/glycolytic muscles such as the EDL possess a more extensive SR network than slow/oxidative muscles like the SOL. Early morphological studies reported that the SR comprises approximately 5.5% of fiber volume in the EDL versus 2.9% in the SOL in mice^[64]^. In addition to greater volume, fast/glycolytic WVL also displays larger terminal cisternae compared to slow/oxidative SOL in guinea pigs^[65]^, contributing to greater calcium handling capacity. Fast/glycolytic EDL muscles exhibit nearly three-fold higher rates of calcium release compared to slow/oxidative SOL muscles of mice^[66]^. This is attributed to a more complex triad architecture and elevated expression of calcium-handling proteins, including ryanodine receptor 1 (RYR1), ATPase sarcoplasmic/endoplasmic reticulum Ca^2+^ transporting 1 (ATP2A1 or SERCA1), and calsequestrin (CASQ). These features have been shown in rat and rabbit muscles, which have compared SOL vs. EDL, superficial vs. deep triceps brachii, and back vs. leg muscles^[65,67–71]^. Recent work has highlighted the importance of selective autophagy of the SR, termed reticulophagy, in maintaining SR homeostasis, particularly under stress or during skeletal muscle remodeling^[72–74]^. Reticulophagy enables the degradation of damaged or surplus SR structures and may serve as a mechanism for dynamically adjusting SR content, which may be important for fiber-specific changes depending on demand. While still underexplored in skeletal muscle, emerging data suggest that reticulophagy could contribute to the turnover and plasticity of the SR network, complementing established morphological and functional differences between fiber types^[72–74]^.

Similar distinctions exist within the mitochondrial network, which is among the most metabolically and structurally diverse organelles in skeletal muscle. Mitochondria are typically organized in two key domains: the subsarcolemmal pool, thought to support transcriptional and signaling functions and occupies 20% of the mitochondrial network, and the intermyofibrillar network, which supplies ATP for contraction and occupies approximately 80% of the mitochondrial network^[75–77]^. In slow/oxidative fibers, the mitochondrial network is more extensive, with volume fractions reaching 10–15% of the fiber volume compared to only 3–5% in fast/glycolytic fibers from rodents^[78–80]^. This is accompanied by greater cristae density, enhanced respiratory efficiency, and a higher degree of network connectivity^[78–81]^. In parallel with structural differences, aspects of mitophagy also appear to differ across skeletal muscles of different fiber type composition. Studies in rodent muscles have shown that predominantly slow/oxidative muscles (e.g., SOL and DIA) express higher levels of mitophagy-related proteins such as BCL2 interacting protein 3 (BNIP3) and parkin (PRKN) compared to fast/glycolytic muscles (e.g., TA, gastrocnemius [GAS], plantaris, and WVL)^[41,44]^. However, findings are not entirely consistent; some studies report greater BNIP3 in fast/glycolytic WVL compared to SOL^[42]^. Moreover, mouse data from our group indicate that despite higher expression of select mitophagy markers, slow/oxidative muscles exhibit lower mitophagy flux (unpublished observations), which may be due to the structural complexity of their mitochondrial network, potentially making organelles more resistant to turnover.

Beyond the SR and mitochondria, organelles such as peroxisomes, lysosomes, and nuclei also likely differ in structure and function across skeletal muscle fiber types^[33,61–63]^. However, our understanding of how these organelles are differentially regulated by autophagy in slow/oxidative versus fast/glycolytic fibers remains limited. Nonetheless, it is important to note that organelle composition is not static. Collectively, these muscle/fiber type specific differences in organelle organization and autophagy highlight the tight integration between intracellular architecture and metabolic phenotype. They also underscore the need for further research to resolve key questions regarding how autophagic pathways selectively target and regulate different organelles across different muscles and fibers.

## Recruitment patterns likely impact fiber-specific autophagy during activity and disuse

Motor unit recruitment patterns critically shape the adaptive responses of skeletal muscle fibers to contractile activity and disuse. Recruitment follows the size principle, whereby smaller, slow-twitch motor units are activated during low-intensity, sustained activity, while larger, fast-twitch motor units are progressively recruited as force demands increase^[82–84]^. This hierarchical activation governs force production and metabolic demands placed on different fibers during activity. For example, low-intensity exercise primarily engages slow/oxidative fibers, favoring FAO and mitochondrial respiration, whereas high-intensity exercise adds by further recruiting fast/glycolytic fibers, increasing reliance on anaerobic glycolysis, and triggering acute energetic stress^[6,9,10]^. These fiber type-specific recruitment patterns result in distinct intracellular environments characterized by varying levels of ATP turnover, calcium dynamics, and reactive oxygen species (ROS) production, all of which are established regulators of autophagy and mitophagy signaling^[7]^. Moreover, recruitment patterns lead to selective substrate utilization and depletion in specific skeletal muscle fiber populations. For example, exercise intensities below ~50% VO₂max predominantly engage slow/oxidative fibers, whereas higher intensities (e.g., >85% VO₂max) increasingly recruit type IIA and IIX fibers^[85]^. As a result, differential substrate utilization/depletion and energetic stress may occur selectively within these activated fibers. Such localized changes in metabolic status, particularly reductions in ATP and increases in AMP/ADP, are well-established signals that trigger autophagy and mitophagy (as discussed in the following section). Nonetheless, given that skeletal muscles differ in their fiber type composition and activation patterns during specific tasks, the autophagic response to activity or inactivity is likely inherently different. Even within a single skeletal muscle, not all fibers are uniformly recruited; regional differences in motor unit activation and fatigue can lead to spatially distinct metabolic and signaling responses^[29]^. This heterogeneity is also evident in disuse models such as hindlimb unloading, immobilization, or denervation where differences in baseline recruitment frequency (e.g., tonic activity of the SOL vs. phasic activity of EDL) result in differential muscle/fiber atrophy and thus likely distinct autophagy dynamics. In inactive skeletal muscle (e.g., immobilized or denervated), ATP can accumulate due to reduced energetic turnover, resulting in increased mitochondrial membrane potential, which enhances the potential for ROS generation^[86]^. In fact, our group and others have shown that ROS production is elevated in immobilized mouse muscles and is accompanied by increased autophagic and mitophagic flux, as well as preferential loss of type IIB fibers^[22,87,88]^. Elevated ROS can activate similar transcriptional regulators as exercise, such as the FOXO family of transcription factors, to promote autophagy^[89–91]^. However, a key distinction lies in the functional outcome: while autophagy induced by contractile activity is generally adaptive (i.e., supporting remodeling and mitochondrial quality control), disuse associated autophagy signaling may represent a maladaptive or compensatory response aimed at clearing damaged or excess cellular components under catabolic stress. Taken together, these observations underscore the importance of considering motor unit recruitment patterns and muscle-specific fiber composition when studying autophagic responses in skeletal muscle. Comprehensive analyses integrating contractile activity, metabolic shifts, and autophagic flux across muscle/fiber types are essential to unravel the complex interplay between physiological demands and organelle turnover.

## Fiber-specific integration of energetic, calcium, and redox signals in autophagy regulation

As previously discussed, skeletal muscle fiber types differ markedly in their intracellular signaling environments due to variations in organelle content, metabolic properties, and recruitment patterns. These features influence the baseline dynamics of key intracellular signals, particularly ATP, calcium, and ROS, that are closely linked to autophagy regulation. Fast/glycolytic fibers exhibit greater fluctuations in energy balance during contraction, characterized by rapid ATP turnover, lower mitochondrial density, and lower oxidative potential^[26,80,92]^. These features make them more susceptible to energetic stress during high-frequency or high-intensity contractions. Such energetic stress results in reduced ATP levels and subsequent activation of AMPK, which promotes autophagy via phosphorylation of ULK1 and inhibition of mechanistic target of rapamycin kinase complex 1 (MTORC1)^[93]^. Calcium dynamics also differ substantially across fibers. Fast/glycolytic muscles, such as the EDL, TA, and psoas exhibit high-amplitude calcium transients, which are attributed to a more elaborate sarcoplasmic reticulum and elevated expression of calcium-handling proteins, including RYR1, CASQ, parvalbumin (PVALB), and ATP2A1, across various animal models including mice, rats, guinea pigs, and rabbits^[65,94–97]^. These calcium spikes can activate downstream effectors including calmodulin kinase (CAMK) and calcineurin, which stimulate autophagy through transcription factors such as transcription factor EB (TFEB) to promote lysosomal biogenesis and support autophagosome formation in mammalian cells^[98–100]^. However, excessive mitochondrial calcium uptake can also lead to dysfunction and activate mitophagy^[101]^, though this process remains incompletely understood in skeletal muscle. Fast/glycolytic fibers also generate transient bursts of ROS and possess lower antioxidant buffering^[102]^. Furthermore, these acute ROS elevations can trigger autophagy via redox-sensitive mechanisms, including oxidation of ATG proteins and activation of kinases such as AMPK, CAMK, mitogen-activated protein kinase (MAPK) in human and mouse skeletal muscle, and mammalian cells^[103–111]^. Collectively, the combination of energetic stress, altered calcium dynamics, and ROS surges likely make fast/glycolytic fibers highly responsive to autophagy-inducing signals like exercise and disuse. In contrast, slow/oxidative fibers maintain a more stable energetic profile, owing to their higher mitochondrial content and greater capacity for FAO^[30]^. This metabolic efficiency supports sustained ATP levels and reduces the likelihood of AMPK activation under basal conditions. Calcium transients in slow/oxidative fibers are smaller in amplitude and longer in duration, reflecting their tonic contractile function. These moderate calcium signals are less likely to activate acute autophagy signaling cascades but may support homeostatic regulation. Similarly, although slow/oxidative fibers generate ROS, levels are lower and more tightly regulated due to robust antioxidant systems, including higher catalase (CAT), glutathione peroxidase (GPX), and superoxide dismutase (SOD) activities in rodent and canine models^[112–121]^. In this context, ROS may act as signaling molecules for basal autophagy and redox homeostasis rather than acute stress triggers. Together, these findings are in alignment with data showing higher autophagy flux in fast/glycolytic compared to slow/oxidative muscles. Furthermore, these distinctions define a fiber type-specific autophagic landscape. However, a deeper understanding of how ATP, calcium, and ROS signaling regulate autophagy across muscle/fiber types is still lacking and is essential for elucidating the mechanisms of skeletal muscle remodeling.

## Skeletal muscle remodeling and autophagy alterations in skeletal muscle physiology and pathology

Skeletal muscle remodeling can occur in physiological and pathological conditions, including aging, injury, non-skeletal muscle diseases, and myopathies. Aging induces a shift toward a slower contractile phenotype, with preferential atrophy of type II fibers in both humans and rodents^[122–130]^. Autophagy markers such as SQSTM1 and LC3-II have been shown to accumulate in aged rat GAS, while increased autophagic flux has been observed in the TA and SOL of aged mice^[131,132]^. These changes coincide with reduced ATP synthesis, mitochondrial content, oxidative enzyme activity, and respiratory capacity, as well as altered mitochondrial network morphology (e.g., fragmentation and cristae organization)^[133–137]^. Furthermore, denervation and other models of nerve injury in rodents result in extensive atrophy of affected skeletal muscles, followed by a shift toward a type IIA phenotype^[12]^. Denervation-induced atrophy has long been shown to be accompanied with significant mitochondrial remodeling including significant changes to mitochondrial content, morphology (e.g., swelling and irregular shape) and function (e.g., respiratory dysfunction and increased ROS)^[88,138–144]^. This remodeling is accompanied by increased expression of autophagy-related genes and proteins, as well as elevated autophagic flux in fast/glycolytic muscles like the TA, EDL, and GAS in mice^[22,23,88]^. Furthermore, non-skeletal muscle diseases also induce significant skeletal muscle phenotypic and autophagic changes. In human VL biopsies from patients with chronic obstructive pulmonary disease (COPD), there is an increased proportion of type II fibers and accumulation of autophagosomes, reflected by elevated LC3-II:I ratios^[17]^. Further, VL muscle biopsies from COPD patients express higher levels of BECN1, ATG7, BNIP3, PRKN, and LC3-II^[13]^. Similar patterns are reported in chronic kidney disease (CKD), where human VL biopsies show increased LC3, SQSTM1, BNIP3, and PRKN^[25]^. In CKD mouse models, the GAS shows elevated *Becn1*, *Atg12*, *Bnip3*, and LC3-II:I, while SQSTM1 is reduced. In this same model there is a selective loss of type I and IIA fibers in the EDL^[20,21]^. Inherited myopathies provide further insight into the interplay between fiber type remodeling and autophagy dysregulation. In Duchenne muscular dystrophy (DMD), type II fibers are highly susceptible to early degeneration as observed in VL muscle biopsies from human patients^[16,24]^. In *mdx* mice, GAS exhibits reduced expression of autophagy and mitophagy regulators including LC3-II, *Becn1*, *Atg13*, *Bnip3*, and *Ulk1*^[14]^. In myotonic dystrophy type 1 (DM1), preferential atrophy of type I fibers leads to a relative expansion of type II fiber area^[19]^, accompanied by reduced LC3-II, LC3-II:I ratio, and SQSTM1 in human VL^[15]^. Similarly, Pompe’s disease results in progressive type II fiber loss and is characterized by reduced autophagy flux. Mouse models of Pompe’s disease display elevated LC3-I and LC3-II in SOL and white gastrocnemius (WG), though autophagosome and ubiquitinated protein accumulation is restricted to WG, suggesting a selective vulnerability that may reflect underlying differences in fiber composition, metabolic profile, or skeletal muscle-specific characteristics^[11,18]^. Collectively, these findings underscore the importance of considering multiple skeletal muscle-specific characteristics when evaluating autophagy in disease models. Variability in autophagic responses may arise from a combination of factors, including the underlying disease pathology and the intrinsic contractile, metabolic, and molecular signatures of the affected skeletal muscles. As such, the interpretation of autophagy-related changes should take into account the distinct physiological context of each skeletal muscle rather than assuming uniform responses across this heterogenous tissue.

## Species and strain-specific determinants of skeletal muscle phenotype and autophagy regulation

Skeletal muscle properties differ markedly between humans and commonly used animal models, particularly rodents. One key difference lies in MYH isoform expression: humans express three isoforms resulting in type I, IIA, and IIX fibers, whereas rodents such as mice and rats express a fourth (type IIB), which is absent in human skeletal muscle^[26,30,34,145]^. This contributes to distinct fiber type distributions, with small mammals like mice possessing a greater proportion of fast/glycolytic fibers (type IIX and IIB), and larger mammals like humans having a higher prevalence of oxidative fibers (type I and IIA). These differences extend beyond isoform expression to include variations in metabolic phenotype. Rodent skeletal muscles generally exhibit higher oxidative capacity and mitochondrial content than human muscles^[26,34,145]^. Even within the same anatomical muscle across species, differences are apparent. For instance, type I fibers comprise approximately 80% of fibers in the human SOL, compared to 97% in rats and only ~30% in mice^[26,146]^. While type I fibers are generally the most oxidative in humans, type IIA fibers have the highest oxidative capacity in mice^[26]^, further complicating between-species comparisons in metabolic phenotype and autophagy. Importantly, metabolic and contractile discrepancy also exists within a species. Fibers sharing the same MYH isoform may differ significantly in mitochondrial content, oxidative enzyme activity, and substrate utilization, depending on the specific muscle group or anatomical location^[26]^. Moreover, strain-specific differences within species can also impact mitochondrial and autophagy-related parameters. For example, one study reported that C57BL/6 mice have higher levels of mitochondrial oxidative phosphorylation (OXPHOS) complexes I, III, and IV, as well as the mitochondrial membrane protein voltage-dependent anion channel 1 (VDAC1) in the SOL and quadriceps (QUAD) compared to FVB mice^[27]^. Similarly, another study identified higher mitochondrial DNA (mtDNA) content in FVB mice compared to BALB and NZW strains^[28]^. In addition, sex-based and interindividual variability in fiber composition and contractile performance has been reported in humans and rodents, further influencing physiological responses and adaptation potential^[147–150]^. For example, women have been shown to have a higher proportion of type I fibers and a lower proportion of type II fibers in the vastus lateralis compared to men^[150,151]^, although this difference is not consistent across all muscles or studies^[152–154]^. These considerations are critical when evaluating autophagic or mitophagic activity, particularly when comparing across different skeletal muscles or translating findings from animal models to humans. Given the close relationship between mitochondrial content, energy metabolism, and autophagy/mitophagy signaling, these findings highlight the importance of genetic background in shaping the basal and inducible regulation of autophagy. Altogether, these species- and strain-specific differences underscore the necessity for cautious interpretation and careful experimental design when investigating autophagic responses in skeletal muscle. Understanding how skeletal muscle phenotype, fiber composition, metabolic profile, species, and genetic background influence autophagy is essential for accurate data interpretation and for improving the translational relevance of findings derived from animal models.

## Conclusion

Skeletal muscle is a highly heterogeneous and adaptable tissue, with variation in fiber contractile type, metabolic phenotype, organelle composition, and recruitment patterns shaping its functional responses to physiological and pathological stimuli. The heterogeneity of this tissue plays a central role in modulating how skeletal muscle fibers respond to various signals. Autophagy is essential for maintaining cellular homeostasis and plays a critical role in preserving skeletal muscle integrity by facilitating organelle turnover and proteostasis. In this review, we emphasized that autophagy is not uniformly regulated across all skeletal muscles or fibers. Rather, its autophagy activity and responses are a sophisticated and intricate phenomenon influenced by fiber/muscle-specific features, including contractile phenotype, metabolic capacity, organelle content/density, and recruitment patterns. Additional layers of complexity arise from basal molecular signaling, disease states, and strain- or species-specific differences, further complicating our understanding of autophagy regulation in skeletal muscle. Studies that fail to account for this heterogeneity risk overlooking critical muscle/fiber type-specific and context-dependent mechanisms. Moving forward, both experimental designs and therapeutic strategies must incorporate the diverse characteristics of skeletal muscle to more accurately define the role of autophagy in skeletal muscle plasticity and to optimize interventions for skeletal muscle-related diseases.

## Abbreviations

AMPK: AMP-activated protein kinase
ATG12: Autophagy related 12
ATG7: Autophagy related 7
ATP2A1: ATPase sarcoplasmic/endoplasmic reticulum Ca^2+^ transporting 1
BECN1: Beclin 1
BNIP3: BCL2 interacting protein 3
CAMK: Calmodulin kinase
CASQ: Calsequestrin
CAT: Catalase
CKD: Chronic kidney disease
COPD: Chronic obstructive pulmonary disease
CS: Citrate synthase
DIA: Diaphragm
EDL: Extensor digitorum longus
FAO: Fatty acid oxidation
FOXO: Forkhead box O
GAS: Gastrocnemius
GP: Gastrocnemius-plantaris
GPX: Glutathione peroxidase
MAP1LC3: Microtubule associated protein 1 light chain 3
MAPK: Mitogen-activated protein kinase
mtDNA: Mitochondrial DNA
MYH: Myosin heavy chain
OXPHOS: Oxidative phosphorylation
PVALB: Parvalbumin
PRKN: Parkin
QUAD: Quadriceps
ROS: Reactive oxygen species
RQ: Red quadriceps
RYR1: Ryanodine receptor 1
SIRT1: Sirtuin 1
SOD: Superoxide dismutase
SOL: Soleus
SQSTM1: Sequestosome 1
SR: Sarcoplasmic reticulum
TA: Tibialis anterior
TFEB: Transcription factor EB
ULK1: Unc-51 like autophagy activating kinase 1
VDAC1: Voltage-dependent anion channel 1
VL: Vastus lateralis
WG: White gastrocnemius
WVL: White vastus lateralis
